# COVID-19 pandemic: A multidisciplinary perspective on the pathogenesis of a novel coronavirus from infection, immunity and pathological responses

**DOI:** 10.3389/fimmu.2022.978619

**Published:** 2022-08-26

**Authors:** Jia Yi, Jiameng Miao, Qingwei Zuo, Felix Owusu, Qiutong Dong, Peizhe Lin, Qilong Wang, Rui Gao, Xianbin Kong, Long Yang

**Affiliations:** ^1^College of Traditional Chinese medicine, Tianjin University of Traditional Chinese Medicine, Tianjin, China; ^2^Research Center for Infectious Diseases, Tianjin University of Traditional Chinese Medicine, Tianjin, China; ^3^Institute of Traditional Chinese Medicine, Tianjin University of Traditional Chinese Medicine, Tianjin, China; ^4^Institute of Clinical Pharmacology of Xiyuan Hospital, China Academy of Chinese Medical Sciences, Beijing, China; ^5^School of Integrative Medicine, Tianjin University of Traditional Chinese Medicine, Tianjin, China

**Keywords:** COVID-19, SARS-CoV-2, pathogenic mechanism, virus infected, immune pathogenesis

## Abstract

Coronavirus disease 2019 (COVID-19), caused by severe acute respiratory syndrome coronavirus2 (SARS-CoV-2), has spread to more than 200 countries and regions, having a huge impact on human health, hygiene, and economic activities. The epidemiological and clinical phenotypes of COVID-19 have increased since the onset of the epidemic era, and studies into its pathogenic mechanisms have played an essential role in clinical treatment, drug development, and prognosis prevention. This paper reviews the research progress on the pathogenesis of the novel coronavirus (SARS-CoV-2), focusing on the pathogenic characteristics, loci of action, and pathogenic mechanisms leading to immune response malfunction of SARS-CoV-2, as well as summarizing the pathological damage and pathological manifestations it causes. This will update researchers on the latest SARS-CoV-2 research and provide directions for future therapeutic drug development.

## 1 Introduction

Since the end of December 2019, a global outbreak epidemic has emerged in several countries as an acute respiratory infection caused by a previously undiscovered strain of coronavirus (1). On December 1, 2019, the first pneumonia of unknown origin was identified in Wuhan, Hubei Province, and confirmed as a new coronavirus on January 8, 2020 (2, 3). On 12 January 2020, the World Health Organization (WHO) tentatively named this virus as “2019 new coronavirus (2019-nCoV)”, and on 11 February, the International Committee on Classification of Viruses officially called it “SARS -CoV-2”, and on the same day WHO unified pneumonia caused by SARS-CoV-2 infection as “Coronavirus disease 2019 (COVID-19)”. This pneumonia is associated with a novel strain of RNA virus from the coronavirus family. In terms of clinical manifestations, COVID-19 has a lower morbidity and mortality rate than SAS and MERS. Still, it spreads faster and more widely, and the number of infections and deaths far exceeds those of the first two viruses (4), which are highly infectious, have a long incubation period, and are prone to mutation (5). COVID-19 can lead to severe acute respiratory infections and multiple organ systems functional impairment, with 15-30% of COVID-19 patients requiring Intensive Care Unit (ICU) admission and organ function support therapy, with an overall morbidity and mortality rate of 4.3%-15% (6), with a morbidity and mortality rate of up to 61% within 28 days in critically ill patients.

Therefore, an in-depth exploration of the pathogenic mechanism of COVID-19 is crucial in achieving an accurate diagnosis, targeted therapy, vaccine development, and improved prognosis. This review provides a detailed overview of the pathogenic mechanism of the new coronavirus based on the pathogenic characteristics of the virus, the process of invasion into the human body, the dysregulation of the immune response caused, and the pathological manifestations and pathological damage of the organism, to provide clinical and scientific assistance.

## 2 Pathogenic characteristics and loci of action of SARS-CoV-2 virus

The main routes of transmission of SARS-CoV-2 are currently considered to be respiratory droplets and close contact (3), with the possibility of aerosol transmission (7) and vertical transmission (8) also present. Data from a clinical study of 1145 patients suggest that the severe course of COVID-19 may be closely related to its viral load during exposure (9). Therefore, studying the pathogenic characteristics of SARS-CoV-2 and its loci of action is essential to understanding the pathogenic mechanisms of the virus.

### 2.1 Pathogenetic characteristics of SARS-CoV-2 virus

SARS-CoV-2 is a member of the coronaviridae family, order Nestoroviridae, genus Pre-coronavirus, and is a spherical enveloped virus with a diameter of approximately 120 nm (10). Mature SARS-CoV-2 viral particles consist of a positive 5’-plus-cap and 3’-polyadenylate single-stranded RNA with a genomic sequence approximately 30,000 bases long, encoding the structural proteins nucleocapsid phosphoprotein (N), membrane glycoprotein (M), envelope (E), spines (S), and nonstructural protein (nsp) (11). Among them, it is mainly the S glycoprotein that mediates viral entry into target cells and the E and M proteins responsible for viral transcription, translation, and assembly.

The S protein consists of S1 and S2 subunits. The S1 subunit consists of the N-terminal structural domain (NTD) and the receptor binding domain (RBD), which is responsible for the direct binding of Angiotensin-converting enzyme 2 (ACE2) (12); the S2 subunit mediates the fusion of the viral envelope with the host cell membrane (13). It was shown that RBD in the SARS-CoV-2 spike-in (S) protein undergoes a specific point mutation, i.e., the asparagine is replaced by tyrosine at position 501 (N501Y) (14), and thus the N501Y S-protein binds more readily to the ACE2 receptor than the original S-protein (15). The S-protein was found to have four amino acid residues inserted at the junction of subunits S1 and S2 (PRRA) (16). This amino acid sequence can be efficiently cleaved by furin and other proteases (17), which reduce the stability of the SARS-CoV-2 S-protein, enhance viral membrane fusion and infection, and promote viral replication (18).

In addition, it has been proposed that S proteins can circulate within the Golgi and promote S protein cleavage and glycosylation, thereby infecting the plasma membrane of cells (19). Another study found that the S1/S2 cleavage site has remained constant during the human evolution of SARS-CoV-2, suggesting that it provides an adaptive advantage for the virus (14). The antiviral activity of chloroquine and its analogues are well established in the fight against SARS-CoV-2 infection (20), and clinical trials have shown that the use of some chloroquine derivatives can achieve viral reduction and improve the efficacy of the infection (21, 22). And laboratory studies have shown that its antiviral effects are attributed to multiple mechanisms, including fighting coronavirus infection by blocking the glycosylation of host receptors (23, 24), inhibiting the processing of S proteins, and suppressing the inflammatory response (25).

### 2.2 Action sites of SARS-CoV-2 virus

ACE2 is a metallocarboxypeptidase of the renin-angiotensin-aldosterone system (RAS) (26). The pathway of SARS-CoV-2 into host epithelial cells was mainly focused on ACE2 (27). Accordingly, it has been found that tetracycline and doxycycline can act as inhibitors of ACE2-peg binding (28).

ACE2 is expressed in over 150 different cell types in all major human tissues and organs, and its expression levels do not vary by age, sex, or race. Immunofluorescence data showed (29) that ACE2 is expressed at higher levels in epithelial cells of the upper respiratory tract, lung, heart, kidney, testis (30), intestine, liver, pancreas, stomach, duodenum, and rectum (31), and the higher levels of ACE2 in the cilia of the nose compared to the bronchi (32) also suggest that the nose may be the initial site of viral invasion and infection.

ACE2 decreases angiotensin II (Ang II) and is a stimulator of Nicotinamide Adenine Dinucleotide Phosphate (NADPH) oxidase (33). It is a key molecule in the body’s resistance to inflammation and oxidative damage in tissues triggered by SARS-CoV-2. After SARS-CoV-2 binds to the ACE2 receptor and begins to enter the cell and fuses with the viral particle-membrane, ACE2 expression will be downregulated (34), and the affinity of angiotensin II is significantly increased during infection, leading to the susceptibility of the virus in binding to ACE2 (35). It was shown that the affinity of SARS-CoV-2 to ACE2 receptor is about 10-20-fold higher than SARS-CoV (36). Therefore, based on the persistent downregulation of ACE2 expression, the overproduction of angiotensin II and activation of NADPH oxidase leads to enhanced oxidative stress mechanisms along with the release of inflammatory molecules (37), leading to the rapid progression of the disease.

In addition, cell surface phospholipid proteoglycans (HSPGs) interact with the S protein of SARS-CoV-2 (18), triggering a conformational change in the S protein RBD, and acting as a cofactor during viral endocytosis (33), which facilitates viral binding to its specific receptor (38). Basic studies suggest that HSPGs are modified by 3-OST isoform 3 but not 3-OST isoform 5, increasing S protein-mediated fusion between SARS-CoV-2 and cells, suggesting a role in virus transmission (39). In particular, HSPGs have been identified as adhesion receptors for SARS-CoV-2 infection in isolated human lung tissue explants from human lung epithelial cell nuclei *in vitro* (40).

**Figure 1:**
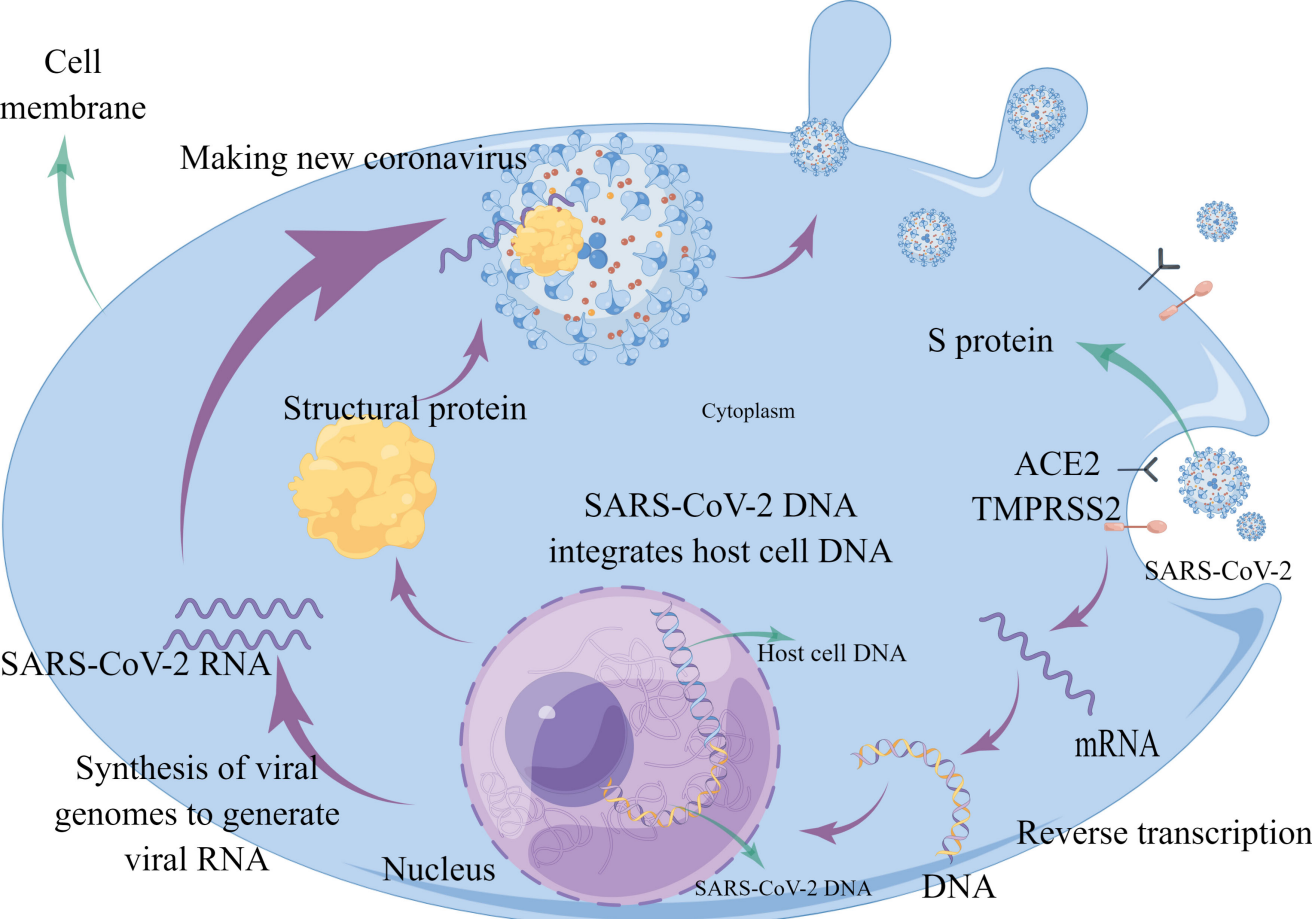
Schematic diagram showing SARS-CoV-2 injecting RNA into the host cell by binding to the ACE2 receptor on normal cells *via* the S protein. The injected RNA uses the nutrients in the host cell to replicate itself and make the structural proteins it needs. The structural proteins combine with the RNA to form a new virus.

RNA sequencing also revealed that immune cells, although not expressing ACE2, are transmembrane proteins of immunoglobulin cluster of differentiation (CD) 147, providing a pathway for the virus to enter and attack immune cells (41–43). It should be noted that one study using single-cell sequencing found that few cells in the placenta express both ACE2 and transmembrane serine proteases (TMPRSS2), thus concluding that ACE2 is not an effective route of transmission from mother to child (44). (**Figure 1**) Also, in combination with the replication process of new coronaviruses, the nucleoside analogue favipiravir (T-705) was found to effectively inhibit the RNA polymerase activity of RNA viruses (45). Remdesivir targets RNA-dependent RNA polymerase (RdRp) and is a nucleotide analogue. Remdesivir received emergency use authorization from the US Food and Drug Administration (FDA) and was approved as the first drug to treat patients with COVID-19 (46). The pharmacological mechanism of the drug is to interfere with the polymerization of viral RNA (47). As a broad-spectrum antiviral drug, it has significant antiviral activity against several RNA viruses such as Ebola virus, coronavirus (48), hepatitis C virus, and human immunodeficiency virus (HIV) (49). Experimental studies have shown that the drug significantly inhibited SARS-CoV-2 virus infection in Vero E6 cells (50), while reducing viral load and pulmonary pathological changes in animal models (51), and had stronger antiviral activity in combination with interferon (52). Lopinavir/ritonavir (LPV/r) are two anti-HIV protein hydrolase inhibitor (PI) drugs that act as antiviral retroviral with a pharmacological mechanism that prevents the excision of the Gag-Poll polyprotein (53), leading to the immaturity of the virus that replicates and proliferates in the organism. Clonidine is also thought to inhibit RNA virus replication by entering the infected cells during viral RNA (54).

At the same time, studies have pointed out that papain-like protease (PLpro) and main protease (Mpro/3CLpro) are two crucial proteases produced by the new coronavirus. Therefore, inhibition of PLPro and Mpro/3CLpro can effectively inhibit virus infection and replication, and is a vital target for antiviral drug development (55). The researchers screened and evaluated the applicability of a batch of FDA-approved clinical drugs targeting PLpro to SARS-CoV-2 PLpro, and found that a naphthalene-based noncovalent inhibitor GRL0617 works by occupying and blocking the PLpro substrate-binding pockets S3 and S4 exerted a potent inhibitory activity. In addition, studies have also identified inhibitors against novel coronavirus Mpro, including boceprevir, GC-376, and calpain inhibitors II and XII, which are often mimetic peptides that mimic natural peptide substrates and covalently bind to residue C145 in Mpro to exert inhibitory effects. (56). And a study selected 47 from the list of 3987 FDA-approved drugs for *in vitro* 3CLpro enzyme inhibitor screening test, and observed that boceprevir, ombitasvir, paritaprevir, tipranavir, and micafungin showed partial inhibition, and ivermectin blocked. The 3CLpro activity of SARS-CoV-2 was more than 85%, indicating that it has the potential to inhibit the replication of SARS-CoV-2. In addition, PF-07321332, developed by Pfizer, is the first oral coronavirus-specific major protease inhibitor approved by the U.S. FDA. The FDA has approved emergency treatment for Paxlovid (PF-07321332 and ritonavir). As a protease inhibitor, PF-07321332 binds to viral enzymes and can block the activity of proteases required for the coronavirus to replicate itself. Ritonavir, an inhibitor of a key liver enzyme called CYP3A, also increased and maintained plasma concentrations of PF-07321332 when given in combination (57).

### 2.3 Mediating factors of SARS-CoV-2 virus invasion into cells

The consensus achieved by the current study is that the entry and spread of the SARS-CoV-2 virus depend on the host ACE2 receptor and the serine protease TMPRSS2, with possible involvement of B/L7 and furin proteases (27).

Experimental studies have shown that the S protein of the SARS-CoV-2 virus binds to the receptor, acid-dependent proteolytic cleavage (58), and is assisted by the S2 subunit to mediate the fusion of the viral membrane with the cell membrane (59), leading to cytoplasmic lysis. This process is mainly mediated by certain host proteases, including furin protease, TMPRSS2, histone B, histone L, factor Xa, and elastase (60). Bertram et al. also suggested that the coronavirus protease system is transmembrane anchored, which is essential for invasion and infection (61). As previously described, after membrane fusion and protease mediation, the S1/S2 site of the S protein will insert four amino acids, providing a motif that can be recognized and cleaved by the furin protease. The virus is then cleaved by TMPRSS2, and the viral protease system forms an unlocking and fusion catalytic structure with the type II transmembrane serine protease (TTSP) family at the cell surface and mediates rapid entry into the cell and completion of ligation within the cell (61, 62), triggering irreversible and extensive conformational changes that mediate membrane fusion (63, 64).

In addition to the associated proteases, it has been proposed that coronavirus infection increases circulating exosomes containing lung-associated autoantigens as well as viral antigens and 20S proteasomes (65). It has also been shown that SARS-CoV-2 drives host cell molecular pathways to activate cellular kinases, such as casein kinase II (CK2) and p38 mitogen-activated protein kinase (MAPK), and growth factor receptor (GFR) signaling to hijack the host protein production machinery (66) for its replication, transcription, and translation purposes.

## 3 Abnormalities in the body’s immune response

When normal, the body’s immune system can limit processes such as the entry of viruses into host cells and their replication within the host cells. The immune system has two main defense mechanisms: innate immunity and adaptive immunity. In contrast, after infection with the SARS-CoV-2 virus, the pathogen-associated molecular patterns (PAMPs) of the virus trigger specific combinations of pattern recognition receptors (PRRs) and adapter molecules, leading to an immune response adapted to the pathogen (67), resulting in abnormal immune response function and causing the associated pathological processes.

### 3.1 Inherent immune response dysregulation

Innate immunity is the first line of defense against infection. The main cells that perform innate immunity are mast cells, NK (natural killer cells), NKT (natural killer T cells), NHC (natural helper cells), granulocytes, macrophages, and monocytes. The organism detects coronaviruses through PRRs, which trigger an innate immune response that effectively limits viral replication. And it helps to control or eliminate viral infections by releasing interferons (IFNs), while activating interferon-stimulated genes (ISGs) to exert direct antiviral effects and recruit antiviral immune effector cells to clear the virus (**Table 1**).

**Table:** Table 1 Summary of research progress on COVID-19 innate immune response dysregulation.

Pathological process	Mechanism	Presenters	Time
Excessive inflammatory response	ROS is associated with reduced numbers and functional failure of NK cells.	Osman; Zheng	2020
	SARS-CoV-2 suppresses immune response and causes infection through activation of Nox2.	Violi	2020
	Binding of PAMPs to PRRs induces intrinsic immune signaling.	Higashikuni	2021
	Immune signaling sequentially involves adaptor proteins (MYD88, TRIF, RGL-1 and MAD-5), cell membrane protein kinases (IRKs, MAPKs and ERKs) and finally transcription factors (e.g. nuclear factor kappa-B, IFRs, NF-κB and IFRs) are produced at the cell membrane.	Vabret	2020
	Transcription factors migrate to the nucleus and induce the expression of encoded cytokines, IFN-I, IFN-III, pro-inflammatory cytokines and chemokines.	de Wit	2016
	CRAC channel inhibitors block the release of pro-inflammatory cytokines and protect the integrity of endothelial cells.	Bruen	2022
Evasion of natural immune system recognition	Nsp16 and nsp10 induce the synthesis of viral mRNAs that mimic host cell mRNAs, thereby protecting the virus from the host intrinsic immune response.	Viswanathan	2020
	SARS-CoV-2 nsp1 causes mRNA translation shutdown in host cells and blocks RIG-I and ISG.	Higashikuni	2021
	SARS-CoV-2 inhibits interferon-induced and blocked IFN signaling and leads to decreased expression levels of toll-like receptor 7, TLR8, TLR2 and TLR4 receptors that recognize SARS-CoV-2 viral RNA, producing immune escape.	V'Kovski	2021
Interferon response dysregulation	The immune evasion mechanism of SARS-CoV-2 is also associated with the inhibition of IFN production and IFN signaling by viral proteins.	Hadjadj; Jiang	2020
	The protease of SARS-CoV-2 can directly cleave IRF3, resulting in diminished IFN production.	Moustaqil	2021
	IRF7 and IRF9 are upregulated in SARS-CoV-2 infection and severe viral load may overwhelm the IFN response and determine the outcome of the infection.	Hasan	2021
	ORF-6 acts as an antagonist of type I interferon promoting viral escape from the host intrinsic immune system.	Fiorino	2021
	Viral proteins or nucleic acids that trigger PRRs induce β-interferon TIR structural domain bridging proteins (TRIFs) and IRFs *via* TIR domain-containing junctions, thereby activating the interferon response.	Prompetchara	2020
	SARS-CoV-2 viral protein's interference with interfering with the production of IFN leads to or blocks the downstream signaling pathway following the binding of IFN to ISGs.	Bastard; Zhang	2020

#### 3.1.1 Excessive inflammatory response

Studies have shown that reduced numbers and functional failure of NK cells occur during SARS-CoV-2 infection (68, 69), and the mechanism may be closely related to the generation of reactive oxygen species (ROS) during the early stages of the immune response. In addition, Nox2 may be a key factor in the infection and development of COVID-19. It was found that the SARS-CoV-2 virus may suppress the immune response and lead to infection by activating Nox2 (NADPH oxidase 2, nicotinamide adenine dinucleotide phosphate hydrogen oxidase 2) (70). Clinical observations likewise revealed higher levels of Nox2 activation in critically ill patients with COVID-19 (37).

**Figure 2:**
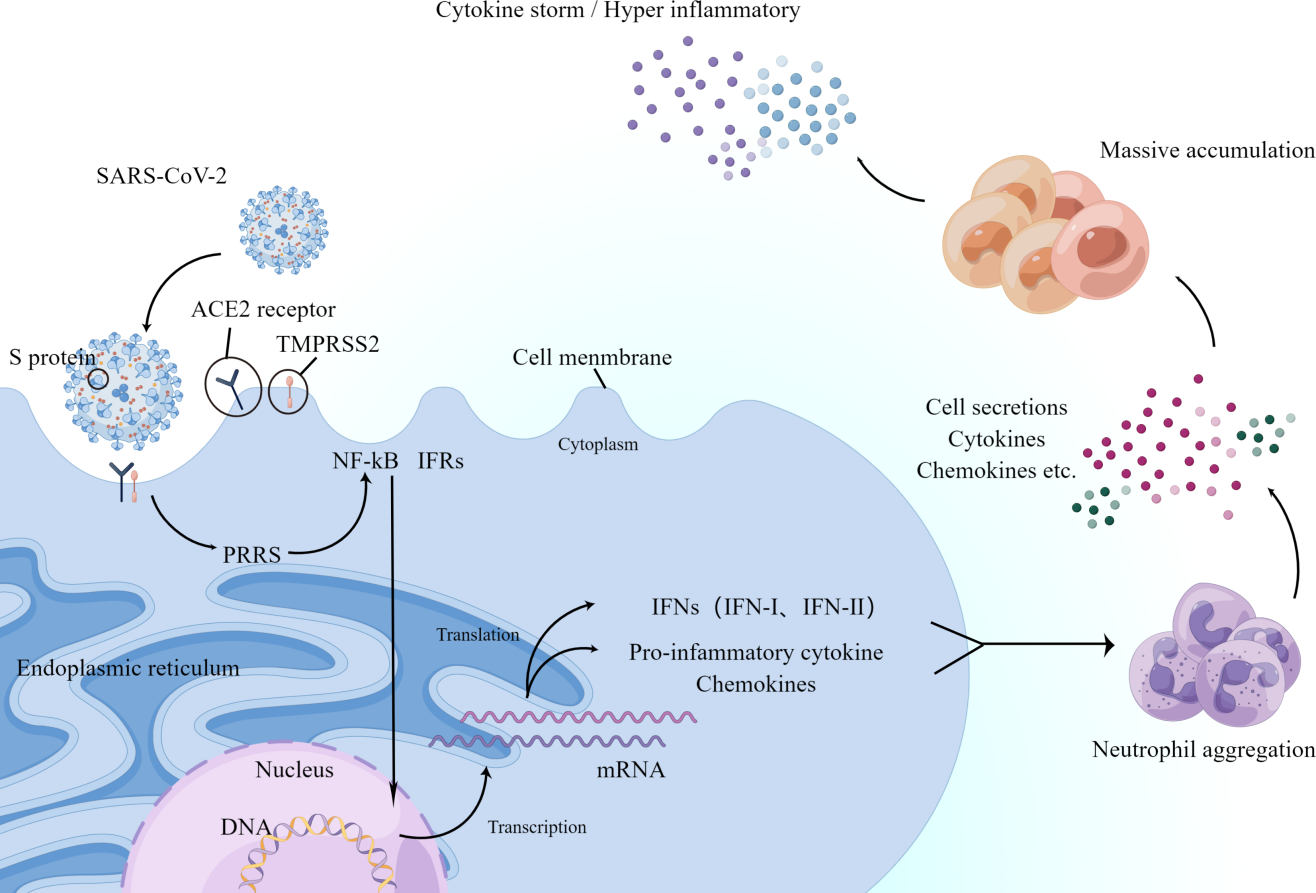
Schematic diagram showing the process by which the new coronavirus enters the human body and triggers an inflammatory response. ACE2 and TMPRSS2 play a decisive role in neo-coronavirus invasion. The major PRRs against viruses are present on the cytoplasmic and endosomal membranes of immune cells and recognize foreign viruses. After a series of processes, they finally produce transcription factors NF-κB and IFRs on the cell membrane. Next, they migrate to the nucleus and induce the expression of encoded cytokines and IFN-I and IFN-III, pro-inflammatory cytokines, and chemokines, which in turn accumulate large numbers of neutrophils. The secretion of neutrophils, cytokines, and chemokines promotes further accumulation of immune cells, producing an excessive inflammatory response or further triggering the cytokine storm mentioned below.

The main PRRs-against viruses are currently considered to be Toll-like receptors (TLRs) and RIG-I-like receptors (RLRRs), and NOD-like receptors (NLRX) (71). Among them, PRRs are present on the cytoplasmic and endosomal membranes of immune cells, and their function is to recognize foreign pathogens on the cell surface or inside. The binding of PAMPs to PRRs induces innate immune signaling (17), successively involving adaptor proteins (MYD88, TRIF, RGL-1, and MAD-5), cell membrane protein kinases (IRKs, MAPKs, and ERKs), and finally the production of transcription factors at the cell membrane (e.g., nuclear factor kappa-B, IFRs, NF-κB, and IFRs) (72). These transcription factors migrate to the nucleus and induce the expression of encoded cytokines, IFN-I, IFN-III, pro-inflammatory cytokines, and chemokines (73), which leads to a massive accumulation of neutrophils (74). Although neutrophils have an antiviral function, their secretions, cytokines, and chemokines promote the accumulation of immune cells and further produce an excessive inflammatory response (75) (**Figure 2**).

Glucocorticoids should be used for a short period of time, as appropriate, in patients with progressively worsening oxygenation indices, rapidly developing imaging, and over-activated inflammatory responses (76), and systemic corticosteroid use is effective in reducing mortality in critically ill patients with COVID-19 (77). The World Health Organization recommends using dexamethasone 6 mg daily for up to 10 days in patients with severe or critical COVID-19 (78). Calcium-release-activated calcium (CRAC) channel inhibitors block the release of pro-inflammatory cytokines, protect endothelial integrity, and may be effective in treating patients with severe COVID-19 pneumonia (79).

#### 3.1.2 Evasion of natural immune system recognition

SARS-CoV-2 viruses possess ways to escape the natural immune system, such as modifying their own viral mRNAs, inducing mRNA translation abnormalities in host cells, and blocking interferons. It was found that nsp16 and nsp10 induce the synthesis of viral mRNAs that mimic host cell mRNAs, thereby protecting the virus from the host’s innate immune response (80). The spike protein of SARS-CoV-2 facilitates invasion of host cells and evades detection by host immune cells. It was found that the nsp1 of SARS-CoV-2 causes mRNA translation shutdown in host cells and blocks Retinoic acid-inducible gene I (RIG-I) and Immune Serum Globulin (ISG), key mediators of the innate immune response against viral infection (17). Furthermore, SARS-CoV-2 will inhibit interferon-induced and blocked IFN signaling and lead to decreased expression levels of toll-like receptor 7, TLR8, TLR2, and TLR4 receptors that recognize SARS-CoV-2 viral RNA, which will lead to immune escape as SARS-CoV-2 virus is not recognized by the host’s immune system (81).

#### 3.1.3 Interferon response dysregulation

IFNs are the first line of defense against viruses. It includes a series of antiviral IFN cytokines, classified into types I, II, and III according to their unique molecular structures, which trigger the expression of ISGs. ISGs exert various antiviral and other immunomodulatory functions by directly inhibiting viral replication (82), transcription, and translation through multiple mechanisms.

It is important to note that viruses (especially those infecting the lung) develop strategies to evade PRR detection and thus alter the host IFN response; for example, some viral proteins can inhibit PRRs in host cells (83). It was found that the immune evasion mechanism of SARS-CoV-2, in addition to the previously described, may also be related to the inhibition of IFN production and IFN signaling by viral proteins (84, 85). Experimental observations revealed that interferon regulatory factor (IRF) mediated signaling was not activated (86), suggesting that dysregulation of interferon response occurs during SARS-CoV-2 virus infection.

Studies suggest that although the organism produces ISGs, transcriptional processes regulated by the interferon regulators IRF3 or IRF7 are apparently absent in SARS-CoV-2 infection. And experiments have shown that the protease of SARS-CoV-2 can directly cleave IRF3 and lead to an attenuated production of IFN (87). Other experiments have shown that IRF7 and IRF9 are upregulated in SARS-CoV-2 infection and that severe viral load may overwhelm the IFN response and determine the outcome of the infection (86), manifesting as a dysregulated IFN response. Studies suggest that Open reading frame 6 (ORF-6) acts as an antagonist of type I interferon, promoting viral escape from the host innate immune system (11).

It was found that viral proteins or nucleic acids that trigger PRRs induce β-interferon TIR structural domain bridging proteins (TRIFs) and IRFs through TIR structural domain-containing junctions, thereby activating the interferon response (88). And triggering PRRs and interferon type I pathway leads to a further oxidative stress response. Meanwhile, the SARS-CoV-2 viral protein has an inhibitory effect on IFN-I-mediated antiviral immune responses. Its interference with interfering with the production of IFN leads to or blocks the downstream signaling pathway following the binding of IFN to ISGs (89, 90), thus antagonizing the innate immune response.

### 3.2 Adaptive immune response dysregulation

When the organism exerts a normal adaptive immune response, the SARS-CoV-2 viral antigen is recognized, processed, and presented by antigen-presenting cells (APCs), thereby activating cellular and humoral immunity. This includes the activation of CD4^+^ and CD8^+^ T cell differentiation. CD4^+^ T cells are activated and differentiate into Th1 and Th2 effector cells and other subpopulations (including Tfh cells, etc.) that recruit immune cells by secreting cytokines (including MIP-1s, INF γ, etc.) and chemokines, CD8^+^ T cells produce substances such as Perforin, CD107a, and Granzyme B, while B cell differentiation and antibody production are stimulated, which together exert adaptive immunity to destroy the virus (58) **(** **Table 2****)**.

**Table 2:** Summary of research progress on COVID-19 adaptive immune response dysregulation.

Pathological process	Mechanism	Presenters	Time
Dysregulated cellular immune response	CD4+ T cells showed significantly reduced responses to various viral proteins such as S, N and M proteins.	Grifoni	2020
	Severely ill patients exhibit macrophage overreaction (also known as macrophage activation syndrome MAS) and lymphocytopenia in effective lymphocytes, including neutrophils, CD4+ T cells, CD8+ T cells and NK cells	Giamarellos-Bourboulis; Schulte-Schrepping; Silvin; Chen	2020
	Lower levels of IFN-g production reduce Th1 production, leading to a further attenuation of the antiviral immune response of CD4+ T cells.	Han	2021
	Th2 cells normally produce IL4, IL-6, Il-8, IL-10 and IL-13, which suppress inflammatory responses and promote antibody responses and inhibit Th1 cell-induced antiviral functions.	Mahlangu	2020
	T-cell lymphopenia may be caused by pro-inflammatory cytokines and activation-induced cell death.	Bellesi; Zheng	2020
Dysregulation of humoral immune response	Helper T cells activate the differentiation of B lymphocytes in the germinal centers of lymph nodes and other lymphoid tissues and secrete pathogen-specific antibodies.	Kumar	2021
	Measurement of serological IgM and IgG titers and detection of SARS-CoV-2 NP antigen by fluorescent immunochromatography showed its high specificity and relatively high sensitivity in the early stages of infection.	Devarajan	2021
	Stalled or delayed synthesis of IgG and IgM antibodies in patients with severe COVID-19	Sun; Wang	2020

#### 3.2.1 Dysregulated cellular immune response

Experimental studies have shown that CD4^+^ T cells are significantly less responsive to various viral proteins such as S, N, and M proteins in SARS-CoV-2 infection (91). Clinical data showed a progressive decrease in peripheral blood CD4^+^ T and CD8^+^ T cells during SARS-CoV-2 infection (92). In contrast, a significant lymphocyte decrease is an important immunological marker of impending severe COVID-19 (93). Severely ill patients exhibit macrophage overreaction (also known as macrophage activation syndrome MAS) and lymphocytopenia in effective lymphocytes, including neutrophils, CD4^+^ T cells, and NK cells (94–96).

It was found that under normal conditions, IFN-g induces the differentiation of Th0 cells into Th1 cells. In contrast, during SARS-CoV-2 infection, lower levels of IFN-g production reduce Th1 production, leading to a further attenuation of the antiviral immune response of CD4^+^ T cells (97). In addition, Th2 cells normally produce IL4, IL-6, Il-8, IL-10, and IL-13, which suppress inflammatory responses, promote antibody responses, and inhibit Th1 cell-induced antiviral functions (98).

In COVID-19 patients, TNF-α and IFN-γ expression is reduced in CD4^+^ T cells (99); high levels of failure markers are expressed in CD8^+^ T cells (100); and programmed cell death protein-1 (PD-1) and T cell immunoglobulin structural domain and mucin structural domain-3 (TIM-3) expression are increased (101). T cells from patients with severe COVID-19 showed high levels of apoptosis and expression of the death receptor FAS (102), suggesting severely impaired T-cell function. It was found that in addition to the reduced number of T cells, the expression levels of T cell receptor subunits, T cell surface molecules, and their downstream signaling molecules were also severely reduced (103). In addition, SARS-CoV-2 infection also resulted in downregulation of B-cell histocompatibility complex MHC-II expression (104), which severely impaired immune function.

It should be noted that since ACE2 is not expressed in T cells, the impaired T cell response may not be due to the direct toxic effects of SARS-CoV-2 (105). It has been suggested that T-cell lymphopenia may be caused by pro-inflammatory cytokines and activation-induced cell death (69, 102).

#### 3.2.2 Dysregulation of humoral immune response

After SARS-CoV-2 infection, cell-mediated immunity of T cells comes into play, and cytotoxic T cells recognize an attack and destroy cells containing this pathogen. Helper T cells activate the differentiation of B lymphocytes in the germinal centers of lymph nodes and other lymphoid tissues to secrete pathogen-specific antibodies (71). Studies have shown that the antibody profile against the SARS-CoV2 virus has a typical pattern of IgM and IgG production. Measurement of serological IgM and IgG titers and detecting SARS-CoV-2 nucleocapsid protein (NP) antigen by fluorescent immunochromatography showed its high specificity and relatively high sensitivity in the early stages of infection (58).

Dysregulated B-cell responses have been reported in COVID-19. Analysis of circulating B cells has shown polyclonal expansion of plasma cells and reduced memory B cells in patients with severe COVID-19 compared to patients with mild COVID-19 or healthy individuals (104, 106, 107), and synthesis of IgG and IgM antibodies also appear to be stalled or delayed (6, 108). Although studies have shown elevated anti-SARS-CoV-2 antibodies in patients with severe COVID-19 (109), their specificity and affinity appear low (110).

## 4 Pathological manifestations of the organism

SARS-CoV-2 viral infection causes disseminated intravascular coagulation (DIC), septic shock (111), RAS system activation, hemodynamic changes, and cellular damage by interfering with the normal function of immune function and triggering cytokine storms and bradykinin storms, which leads to a series of pathological manifestations in the organism.

### 4.1 Cytokine storm

Cytokine storm, also known as cytokine release syndrome, is a potentially fatal immune disease. It is characterized by the high activation of immune cells and the overproduction of large amounts of inflammatory cytokines and chemical mediators (112). It has been proposed that cytokine storm, the excessive immune response that SARS-CoV-2 infection triggers in severe cases of COVID-19 (113), is thought to be a major cause of severe disease and death in COVID-19 patients (74). Cytokine storms begin with strong activation of cytokine-secreting cells (41), and COVID-19 cytokine storms are characterized by high expression of IL-6 and TNF-α (114). The mechanism of which may be related to SARS-CoV-2 induction of cell death and thus histone release, which triggers the secretion of pro-inflammatory molecules of the interleukin-1 (IL-1) family (115), producing such IL-6, IP-10, MIP1αβ (macrophage inflammatory protein-1αβ) and MCP1 (monocyte chemotactic protein-1), and a large number of other pro-inflammatory cytokines and chemokines (116). Among them, IL-6 is an important pleiotropic pro-inflammatory mediator and a major driver of the cytokine storm. And cytokine storm is closely associated with macrophage activation syndrome (MAS). Excessive proliferation of differentiated macrophages leads to phagocytosis and hypercytosis (117, 118), which leads to systemic inflammatory abnormalities.

In addition, CD4^+^ T lymphocytes rapidly differentiate into pathogenic T helper (Th)1 cells that produce IL-6 and GM-CSF (Granulocyte-macrophage Colony Stimulating Factor). GM-CSF plays an important role in mediating the cytokine storm (119). Subsequent induction of high levels of IL-6 and GM-CSF secretion by CD14^+^, CD16^+^, and monocytes (120) exacerbates the cytokine storm. Activated neutrophils can form neutrophil extracellular traps (NETs) that are involved in the pathogenesis of aseptic and nonsterile inflammation (121) and promote the development and progression of inflammation. Uncontrolled excessive inflammatory responses produce oxidative stress (imbalance between oxidants and antioxidants). Activated neutrophils and macrophages release pro-oxidant factors such as TNF-α (tumor necrosis factor-α) and release reactive oxygen species (ROS) (122–124), which in turn stimulate further cytokine production by inflammatory cells, leading to an even more intense inflammatory response (125).

In the face of the inflammatory storm generated by neo-coronavirus, it was found that timely application or combination of monoclonal antibodies can effectively reduce the rate of deterioration and mortality of neo-coronavirus pneumonia, which has broad clinical application prospects. As monoclonal antibodies against interleukin 6 receptor (IL-6), among which tocilizumab and satralizumab whose pharmacological mechanism is mainly to specifically bind to IL-6 receptor and inhibit its activation, thus inhibiting cytokine storm and reducing mortality (126), clinical studies have also confirmed that the application of this drug has significant efficacy in improving the inflammatory response in patients with COVID-19 (127). In addition, it has been shown that the administration of levilimab in patients with SARS-CoV-2 pneumonia in the absence of other signs of active infection, with or without oxygen therapy, increases the rate of sustained clinical improvement (128), that itolizumab significantly reduces the severe consequences caused by cytokine release syndrome (129), and that tocilizumab reduces the duration of hospitalization (130), the progression to mechanical ventilation (131) and the risk of transfer to the ICU (132). In addition, studies have proposed binding neutralizing antibodies to the surface of photothermal nanoparticles (NPs) to capture and inactivate novel coronaviruses. The NPs consist of a semiconductor polymer core and a biocompatible polyethylene glycol surface modified with a high-affinity neutralizing antibody. The multifunctional NP efficiently captures novel coronavirus pseudoviruses and completely blocks virus infection of host cells *in vitro* by surface-neutralizing antibodies. In addition to the virus capture and blocking functions, the NPs have a photothermal function to inactivate the virus by generating heat upon irradiation (133). The multifunctional nanoparticles also exhibit excellent biosafety *in vitro* and *in vivo*, and show satisfactory pulmonary delivery in mice. Most importantly, *in vivo* treatment with multifunctional NPs in the presence of actual novel coronaviruses was achieved, offering significantly improved therapeutic efficacy compared to soluble neutralizing antibodies and demonstrating their great potential for clinical novel coronavirus therapy. NPs are very superior to neutralizing antibodies in the treatment of actual novel coronavirus infections that occur *in vivo*. This versatile NP provides a flexible platform that can be easily adapted to other novel coronavirus antibodies and extended to new therapeutic proteins, and thus it promises to provide broad protection against the original novel coronavirus and its variants (134).

### 4.2 Coagulation disorders

Clinical observations have revealed alterations in hematology associated with coagulation during COVID-19 (2, 135). In most severe cases, patients develop microvascular dysfunction such as disseminated intravascular coagulation (DIC) or infectious shock (136). Thromboembolic complications are one of the main causes of morbidity and mortality in patients with COVID-19 (137).

The cause of thrombosis is an imbalance between procoagulant and anticoagulant processes. Systemic thromboembolism, including venous thromboembolism, arterial thrombosis, and thrombotic microangiopathy, is a unique and essential feature of COVID-19. In current studies, the mechanisms of coagulation disorders may also be associated with downregulation of ACE2 activity, endothelial dysfunction (138), activation of von- Willebrand factor, activation of the complement system, neutrophil extracellular traps (139), oxidative stress injury, and high inflammatory state (140) formation. These predisposes infected individuals to the activation of Virchow’s triad, leading to arterial and venous thrombosis and vascular arrest anywhere in the body (141).

It has been studied that coagulation parameters, especially D-dimer levels, predict mortality in 2019 coronavirus disease and that patients with 2019 coronavirus disease have an increased risk of arterial and venous thrombosis. It has been suggested that anticoagulation (AC) is beneficial in these patients. That prophylactic AC with enoxaparin and apixaban is appropriate for treating hospitalized 2019 coronavirus disease patients with D-dimer levels >1µg/mL (142).

#### 4.2.1 Down-regulation of ACE2 activity

As mentioned previously, the primary site of action of the SARS-CoV-2 virus is ACE2. And viral infection may lead to a decrease in ACE2 activity, resulting in elevated angiotensin II and decreased angiotensin 1-7. Angiotensin II rapidly generates reactive oxygen species mediated by NADPH oxidase and causes oxidative stress injury (143). Angiotensin 1-7 is now considered to be an important anti-inflammatory and anti-thrombotic peptide with inhibitory effects on platelet activation (144). Therefore, these will lead to RAS dysregulation, oxidative stress injury, and coagulation disorders.

#### 4.2.2 Endothelial dysfunction

There is a strong correlation between endothelial dysfunction and thrombosis (145). Experimental studies have shown that endothelial dysfunction is a key factor in the release of the procoagulant factor fVIII (146) to generate and activate thrombus and trigger various coagulation cascades (147). This process may be associated with the endothelial expression of many prothrombotic factors and receptors. In addition, overexpression of hemagglutinin-like oxidized low-density lipoprotein receptor (LOX-1), cyclooxygenase (COX-2), and vascular endothelial growth factor (VEGF) during infection can also cause endothelial injury (148).

#### 4.2.3 Activation of the von Willebrand factor

The underlying vascular hemophilic factor (vWF) plays a key role in COVID-19-related coagulation (149). Following endothelial injury, vWF present in the subendothelium is released, further multimerized by disulfide bonds, and exposes to the platelet-binding and collagen-binding domains (150). vWF acts as an adherent molecular glue platelet together with subendothelial collagen, activating platelet aggregation and thrombosis (151).

#### 4.2.4 Activation of the complement system

The complement system is capable of activating the coagulation cascade through multiple mechanisms leading to vascular thrombosis. The nucleocapsid (N) protein of SARS-CoV-2 binds to mannose-binding lectin-associated serine protease (MASP)-2, which is expressed on microvessels, leading to complement activation (121). In contrast, complement factor C3 and MAC directly activate platelets and induce platelet aggregation (152). Similarly, complement factor C5a has been shown to stimulate the expression of fibrinogen activator inhibitor 1, thereby promoting thrombosis (153).

#### 4.2.5 Formation of neutrophil extracellular traps

NETs, also known as extra-neutrophil traps, contain various pro-thrombogenic molecules such as tissue factor, protein disulfide isomerase, factor XII, vWF, and fibrinogen (154). In addition, DNA released from extracellular NETs can directly activate platelets and lead to thrombosis. Circulating histones (major components of NETs) have also been found to activate Toll-like receptors on platelets and promote thrombin production (155).

### 4.3 Other pathological manifestations

Coronavirus replication can lead to lysosomal disruption, mitochondrial damage, free radical damage, disruption of membrane structure and function, destruction of mitochondria and lysosomes, cellular autolysis, and triggering ion concentration imbalance (73). Among them, reactive oxygen species (ROS) and (reactive nitrogen species) RNS may be one of the modification pathways of severe COVID-19 (156). It has been demonstrated that the downregulation of ACE2 by COVID-19 may affect the mitochondrial function of immune cells, which in turn may reduce the immune function of the host (157).

As mentioned earlier, ACE2 is an important locus for the SARS-CoV-2 virus. And one of the roles of ACE2 is to inactivate angiotensin II by converting it to angiotensin 1-7 through proteolysis, which puts ACE2 in a critical position to act as a negative regulator of the renin-angiotensin-aldosterone system (RAAS) (158) and leads to RAAS system dysfunction.

## 5 Pathological damage to the organism

Infection with SARS-CoV-2 will cause a range of pathological injuries such as lymphopenia, and lung tissue damage (159), such as acute respiratory distress syndrome (ARDS) and respiratory failure, sepsis-induced cardiac injury and arrhythmia (58), and multi-organ failure. Enhanced granulocyte and monocyte-macrophage infiltration are common in critically patients with COVID-19. Monocytes and macrophages are involved in and exacerbate hypersensitivity reactions (160), leading to organ damage.

### 5.1 Lymphoid tissue damage

Studies have shown a direct relationship between apoptosis rates and the pathogenicity and severity of COVID-19 (161). COVID-19 attacks the lymphoid tissue of the body and induces apoptosis in immune cells. During SARS-CoV-2 infection, single-cell RNA sequencing showed enrichment of SARS-CoV-2 RNA in the macrophage population of bronchoalveolar lavage samples from patients phenomenon, suggesting that the virus directly infects and attacks macrophages (162) and triggers macrophage polarization toward a pro-inflammatory phenotype (163).

Several mechanisms may exist for apoptosis, decreased expression, and functional failure of immune cells. The decrease in T-cell numbers was negatively correlated with IL-6 and TNF-α levels (164), suggesting that increased inflammatory cytokines may promote T-cell failure and apoptosis. Moreover, the IL-2 signaling pathway is inhibited, negatively regulating CD8^+^ T cells (71) and inducing a decrease in lymphocytes. Besides, some lymphoid organs are attacked by SARS-CoV-2, which further leads to lymphocyte damage. Similarly, it has been noted that SARS-CoV-2 ORF3a induces apoptosis through the extrinsic apoptotic pathway. Caspase-8 activation/cleavage is a hallmark of the extrinsic apoptotic pathway, and SARS-CoV-2 ORF3a induces caspase-8 activation/cleavage. This process can induce epithelial apoptosis and inflammatory cytokine processing in turn, which triggers necroptotic prolapse pathway caspase-8-mediated apoptotic activation and inflammatory response, which can induce downstream immunopathogenesis in lung tissue (165).

Furthermore, elevated blood lactate levels in critically ill patients with COVID-19 inhibit lymphocyte proliferation of neutrophils with suppressive properties (e.g., granulocyte myeloid-derived suppressor cells (G-MDSCs)) (166). They may inhibit the expansion of CD4^+^ and CD8^+^ T lymphocytes (167).

### 5.2 Diffuse lung injury

Preliminary data suggest that pulmonary vascular injury and partial loss of alveolar group function are key to developing severe illness and death in patients with COVID-19 (168). Clinical data analyzed that after infection with SARS-CoV-2, most patients develop bilateral interstitial pneumonia with histology showing alveolar wall edema, protein exudates, and non-cellular focal reactive hyperplasia with vascular congestion (169), which also leads to selective death of type II pneumocytes (170). After type II pneumocyte injury, the inflammatory state will be supported by macrophage pro-inflammation (M1), cytokine release, and NF-κB support, further damaging alveolar cells in a vicious cycle (171). Loss of lung surface active gas exchange and vascular abnormalities can lead to progressive respiratory failure. Pneumonia caused by SARS-CoV-2 leads to a rapid decrease in arterial pO2 levels measured by transcutaneous saturation (136) and hypoxemia.

Some scholars have suggested that lung tissue damage may be associated with the occurrence of NETs in large numbers of neutrophils, which in turn release toxic enzymes such as elastase (172), and secrete substances such as cationic histones. These, in addition to having direct cytotoxic effects, may also enhance infection of lung cells and thus aggravate the disease (173). In addition, oxidized phospholipids in macrophages triggering cytokine production *via* TLR4-TRIF-TRAF6 can further aggravate lung inflammation (174).

**Figure 3:**
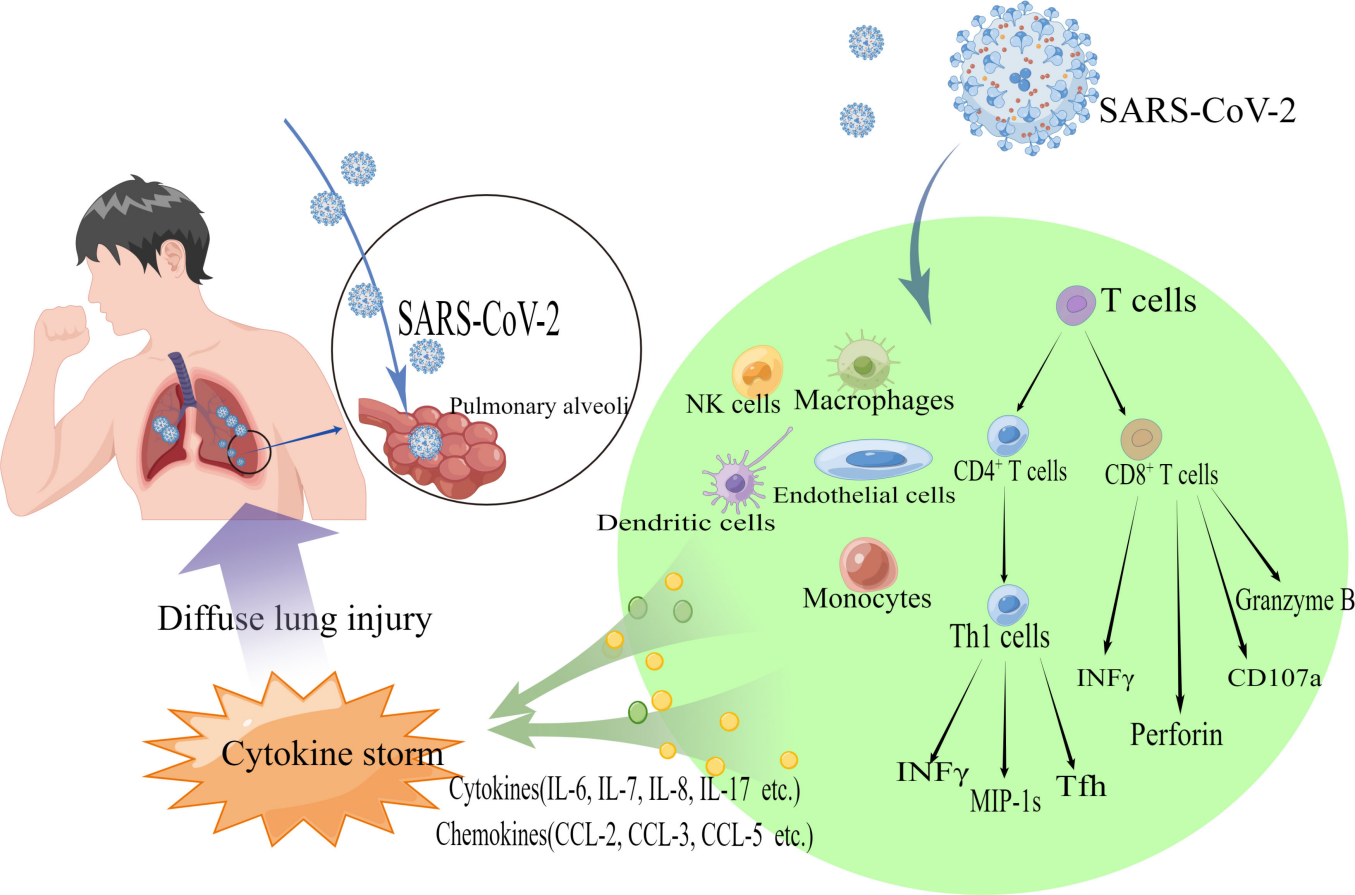
Schematic diagram showing the pathogenic mechanism of diffuse lung injury caused by 2019-nCoV. SARS-CoV-2 binds to ACE2 receptors on human alveolar epithelial cells *via* S proteins and enters the cells. NK cells, natural killer cells, macrophages, dendritic cells, monocytes, etc., release cytokines (e.g., IL-6, IL-7, IL-8, IL-17, etc.) and chemokines (e.g., CCL-2, CCL-3, CCL-5, etc.). CD8^+^ T cells secrete substances such as Perforin, CD107a, and Granzyme B. CD4^+^ T cells are activated and differentiate into Th1, Th2 effector cells, and other subpopulations (including Tfh cells, etc.), and also secrete cytokines (e.g., INFγ) and chemokines to recruit Immune cells are also secreted (e.g., INFγ) and chemokines are recruited, resulting in a cytokine storm that causes diffuse lung injury.

It has been found that patients with ARDS and extrapulmonary complications have significantly elevated rates of circulating pro-inflammatory cytokines, chemokines, and systemic inflammatory markers (175), suggesting that the organism is in a state of intense inflammatory response. It has been proposed that severe lung injury in COVID-19 patients is thought to result from direct viral infection and immune hyperactivation (114). (**Figure 3**)

One study reported that phototherapy using red and near-infrared light reduced lung inflammation and pulmonary fibrosis in mice by downregulating pro-inflammatory cytokines, upregulating IL-10 secretion from fibroblasts and lung cells, and reducing collagen deposition in the lung (176). Since lung inflammation and pulmonary fibrosis are common complications in critically ill patients with novel coronavirus infections, experiments have shown that 650 nm light-emitting diode (LED) treatment may alleviate these life-threatening problems. Compared to conventional laser excitation, 650 nm LEDs have more desirable safety properties. Under its excitation, multifunctional NPs can further inactivate the virus by assisting the photothermal function. In addition, multifunctional nanoparticles have favorable properties for pulmonary delivery and retention, which can overcome the limitation of rapid clearance of antibodies in the lung (177). The unique design of multifunctional NPs not only enables antibody-mediated neutralization to capture novel coronaviruses, but also provides a strategy to mitigate the potential risk of antibody-dependent enhancement (ADE) and new, more infectious novel coronavirus variants by inactivating the virus through direct heating. Together with efficient viral inactivation capabilities, the superior therapeutic efficacy of multifunctional NPs could be further enhanced. Future research will be conducted using site-specific binding approaches, such as site-selective click chemistry (178) that can increase surface antibody binding efficiency, control antibody binding sites and orientation and purify multifunctional nanoparticles to improve their therapeutic efficacy further.

### 5.3 Multiple organ injury

According to studies, COVID-19 has been shown over time to cause multi-system involvement of the cardiovascular system (2, 6, 179), the nervous system, the urinary system (2020), and the hematological system in addition to the respiratory and immune systems (139, 180). This is partly due to the widespread expression of ACE2 as a SARS-CoV-2 receptor in tissues and organs, and partly because SARS-CoV-2 causes a series of systemic pathological manifestations, such as cytokine storm and disorders of coagulation mechanisms (74, 181, 182).

In particular, neurological complications have become an increasingly recognized cause of morbidity and mortality in patients with COVID-19. The most common of these neurological symptoms include cerebrovascular events (183), encephalitis, Guillain-Barré syndrome, acute necrotizing encephalopathy, hemophagocytic lymphoid tissue hyperplasia, and acute ischemic cerebrovascular syndrome (184), as well as neuropsychiatric symptoms such as dizziness, sleep disturbances, cognitive deficits, delirium, hallucinations, and depression. In addition, the chronic neurological aspects of traumatic brain injury, post-stroke syndrome, long COVID-19, intractable Lyme disease, and influenza encephalopathy have close pathophysiological similarities, mainly involving positive feedback loops for TNF maintenance and activation (185), and cerebral venous sinus thrombosis (CVT) formation is also associated with infection with SARS-CoV-2 virus (139).

In addition, right ventricular (RV) dysfunction is common and correlates with poor prognosis in COVID-19 patients (186). And one experiment found that ACE2 expressed by enterocytes derived from human colon differentiation is sensitive to SARS-CoV-2 infection, revealing that IFN-c is a strong driver of epithelial cell differentiation towards the enterocyte lineage and leads to high ACE2 expression and increased susceptibility to SARS-CoV-2 (187).

In summary, the pathogenic mechanism of COVID-19 involves the combined effects of characteristic structures, genes, enzymes, and immune responses. The pathological manifestations and damage caused are manifested as a process in which the lung is the main object of damage and can cause extensive extrapulmonary partial damage as the disease progresses.

## 6 Conclusions and challenges

Studies on the pathogenic characteristics of neocoronaviruses and the sites of action have demonstrated that among all functional proteins of neocoronaviruses, the S protein is the main antigenic component that binds to host cell receptor proteins, promotes viral invasion of host cells, and stimulates host immune responses. ACE2 is the main receptor infection target. Serine proteases, cysteine proteases, lysosomal proteases, and other enzymes are the main proteases that activate S proteins. Therefore, the S protein can be selected as an important target for vaccine development. For clinical treatment, ACE direct injection can increase the expression of recombinant ACE2 protein and therapeutic vectors that deliver an expression of high levels of ACE2, which is used to overcome virus-induced ACE2 deficiency by increasing the expression of ACE2 protein. Alternatively, ACE inhibitors can balance ACE/ACE2 function by inhibiting the activation of S proteins through the inhibition of related enzyme activities (188). The study of viral exosomes can also be further explored to explore the possible target proteins and signaling molecules for exosomal-cell fusion, thus obtaining new ways to inhibit virus transmission.

During the immune system dysregulation caused by a new coronavirus, the virus modifies its RNA, generates an immune escape mechanism, antagonizes the immune response, and induces apoptosis of immune cells. This phenomenon suggests that viruses change by adapting to new environments, and it is speculated that a new epidemic involving coronaviruses may break out in the future. COVID-19 is highly infectious and has a long incubation period, resulting in a rampant epidemic with a complex and variable disease course, which poses a great challenge to the control of the epidemic. Up to now, no targeted vaccine or effective drug has been developed.

According to the pathogenic mechanism of neo-coronavirus, treatment is still mainly based on antiviral, anti-infective, and symptomatic supportive therapy. Many drugs have been introduced for the treatment of neo-coronavirus pneumonia; for example tetracycline and doxycycline can act as inhibitors of ACE2 binding (28); niclosamide can inhibit RNA viruses during the post-entry phase of viral RNA replication and also exhibits anti-inflammatory activity (189); chloroquine reduces the production of cytokines and damage-associated molecular patterns by interfering with the innate immune pathways of multiple immune cells, thus preventing experimental sepsis and infectious shock (190, 191). In addition, combination therapies have been introduced, such as early triple antiviral therapy with interferon beta-1b, lopinavir-ritonavir, and ribavirin, which shortens the time to viral shedding, reduces cytokine responses, relieves symptoms, and promotes the discharge of patients with mild to moderate COVID-19 (192). The discovery and study of the pathogenic mechanism of neocoronaviruses can bring the course of action of existing drugs into more explicit articulation at the cellular or molecular level to align with clinical applied medicine with more rationalized, standardized, institutionalized, and scientific research. In addition, we believe that a large number of experiments and research records can provide detailed statistics on drug efficacy, efficiency, and the number of stable cases, which can also help promote the organic combination of pathogenesis research and clinical drug action process analysis. We also suggest that a series of accurate and standardized target models may be constructed, and the criteria for clinically effective associations may be derived through model experiments. The establishment of model systems may be expected to be a reference for subsequent studies on preventing and treating other large epidemic diseases.

SASRS-CoV-2 is another highly lethal virus after SARS-CoV and MERS-CoV. COVID-19 epidemic is a major public health emergency in China and the world. The epidemic is spreading globally and the situation has been dire so far. Further experimental, as well as clinical solutions to the currently unresolved and controversial issues, are still needed. Understanding the pathogenesis of neocoronaviruses and developing therapeutic regimens based on the complex pathogenesis of the pathological damage produced by neocoronaviruses is of great importance in response to COVID-19 and possible future epidemic viruses.

## Author contributions

Conceptualization, JY, XK, QZ and FO; writing—review and editing, JY, JM, QD, QW and PL; visualization, QZ; supervision, XK and LY; project administration, RG, LY. All authors have read and agreed to the published version of the manuscript.

## Funding

This research was funded by the Scientific research project of Tianjin Education Commission, grant number 2021KJ134, Tianjin Municipal Education Commission Scientific Research Project, grant number 2019ZD11, Science and Technology Program of Tianjin, China (21ZYJDJC00070), the National Key Research and Development Program of China (2019YFC1708803), and Innovation Team and Talents Cultivation Program of National Administration of Traditional Chinese Medicine (ZYYCXTD-C-202203), Scientific and technological innovation project of China Academy of Chinese Medical Sciences (Grant No. C12021A04701 to RG)

## Acknowledgments

We thank all the authors of the original work and reviewers for their time and kindness in reviewing this paper. The figures were drawn by Figdraw (https://www.figdraw.com/static/index.html#/).

## Conflict of interest

The authors declare that the research was conducted in the absence of any commercial or financial relationships that could be construed as a potential conflict of interest.

## Publisher’s note

All claims expressed in this article are solely those of the authors and do not necessarily represent those of their affiliated organizations, or those of the publisher, the editors and the reviewers. Any product that may be evaluated in this article, or claim that may be made by its manufacturer, is not guaranteed or endorsed by the publisher.
